# Targeting ST18-mediated pathomechanism in pemphigus vulgaris through voltage-dependent anion channel inhibition

**DOI:** 10.1093/skinhd/vzaf107

**Published:** 2026-01-19

**Authors:** Sari Assaf, Ofer Sarig, Rawaa Ishtewy, Yazeed Zoabi, Yarden Feller, Kiril Malovitski, Janan Mohamad, Shir Bergson, Carmel Bilu, Varda Shoshan-Barmatz, Noam Shomron, Dan Vodo, Liat Samuelov, Eli Sprecher

**Affiliations:** Division of Dermatology, Tel Aviv Sourasky Medical Center, Tel Aviv, Israel; Grey Faculty of Medicine and Health Sciences, Tel Aviv University, Tel Aviv, Israel; Division of Dermatology, Tel Aviv Sourasky Medical Center, Tel Aviv, Israel; Division of Dermatology, Tel Aviv Sourasky Medical Center, Tel Aviv, Israel; Grey Faculty of Medicine and Health Sciences, Tel Aviv University, Tel Aviv, Israel; Grey Faculty of Medicine and Health Sciences, Tel Aviv University, Tel Aviv, Israel; Division of Dermatology, Tel Aviv Sourasky Medical Center, Tel Aviv, Israel; Grey Faculty of Medicine and Health Sciences, Tel Aviv University, Tel Aviv, Israel; Division of Dermatology, Tel Aviv Sourasky Medical Center, Tel Aviv, Israel; Grey Faculty of Medicine and Health Sciences, Tel Aviv University, Tel Aviv, Israel; Division of Dermatology, Tel Aviv Sourasky Medical Center, Tel Aviv, Israel; Grey Faculty of Medicine and Health Sciences, Tel Aviv University, Tel Aviv, Israel; Division of Dermatology, Tel Aviv Sourasky Medical Center, Tel Aviv, Israel; Grey Faculty of Medicine and Health Sciences, Tel Aviv University, Tel Aviv, Israel; Division of Dermatology, Tel Aviv Sourasky Medical Center, Tel Aviv, Israel; Division of Biotechnology, Ben-Gurion University of the Negev, Beer Sheva, Israel; Grey Faculty of Medicine and Health Sciences, Tel Aviv University, Tel Aviv, Israel; Division of Dermatology, Tel Aviv Sourasky Medical Center, Tel Aviv, Israel; Division of Dermatology, Tel Aviv Sourasky Medical Center, Tel Aviv, Israel; Grey Faculty of Medicine and Health Sciences, Tel Aviv University, Tel Aviv, Israel; Division of Dermatology, Tel Aviv Sourasky Medical Center, Tel Aviv, Israel; Grey Faculty of Medicine and Health Sciences, Tel Aviv University, Tel Aviv, Israel

## Abstract

**Background:**

Pemphigus vulgaris (PV), a severe mucocutaneous blistering disease, results from autoantibody-mediated destabilization of epidermal cell–cell adhesion. A functional risk variant at the ST18 locus was found to promote epidermal ST18 expression. Increased ST18 expression was found to aggravate the deleterious effect of PV autoantibodies in part through induction of p53-mediated proapoptotic pathways. The voltage-dependent anion channel (VDAC) is a key regulator of mitochondria-mediated apoptosis.

**Objectives:**

To delineate the interplay between ST18 and VDAC in apoptosis regulation, and the therapeutic potential of VDAC inhibitors in PV.

**Methods:**

We used global RNA sequencing (RNAseq) of human keratinocytes to assess ST18-dependent changes in *VDAC1*, *VDAC2*, *VDAC3* and *BCL2* expression. Immunostaining of skin biopsies was used to evaluate VDAC1 in patients with PV. Apoptotic activity was analysed by caspase 3/7 and TUNEL apoptosis assays, while immunoblotting and a luciferase reporter assay assessed Bcl-2 and p53 pathways. The dispase dissociation assay was used to ascertain the effect VDAC inhibition had on acantholysis.

**Results:**

Keratinocytes overexpressing ST18 showed upregulation of *VDAC1*, *VDAC2* and *VDAC3*, which encode VDAC, and downregulation of *BCL2*, which encodes the antiapoptotic protein Bcl-2. Of interest, mitochondrial VDAC and p53 antagonize Bcl-2 activity. Patients with PV had dramatically increased epidermal VDAC1 expression. Keratinocytes exposed to AK23, a pathogenic antidesmoglein 3 antibody, and overexpressing ST18, exhibited elevated apoptotic activity. VBIT-12, a VDAC oligomerization inhibitor, robustly attenuated this response and concomitantly led to upregulation of Bcl-2 and to downregulation of p53 transcriptional activity. This suggested that inhibition of VDAC ­proapoptotic activity may prevent cell–cell disadhesion in PV. Indeed, VBIT-12 was found to efficiently prevent acantholysis due to PV IgG/AK23.

**Conclusions:**

Our findings identify VDAC as a novel factor in the pathogenesis of PV and thus as an innovative and attractive therapeutic target for the treatment of this disease.

## Teaser

Pemphigus vulgaris (PV), a severe mucocutaneous blistering disease, results from autoantibody-mediated destabilization of epidermal cell–cell adhesion. A functional risk variant at the ST18 locus was found to promote epidermal ST18 expression. Increased ST18 expression was found to aggravate the deleterious effect of PV autoantibodies in part through induction of p53-mediated pro-apoptotic pathways. Here we investigated the role of voltage-dependent anion channel (VDAC), which is a key regulator of mitochondria-mediated apoptosis in ST18-mediated PV pathogenesis. Our results suggest that inhibition of VDAC pro-apoptotic activity may prevent cell–cell disadhesion in PV, pointing at VDAC inhibition as an innovative and attractive approach for the treatment of this disease.

What is already known about this topic?

- Pemphigus vulgaris (PV), a severe mucocutaneous blistering disease, results from autoantibody-mediated destabilization of epidermal cell–cell adhesion.
- Increased ST18 expression aggravates the deleterious effect of PV autoantibodies through induction of p53-mediated proapoptotic pathways.
- Previous data have highlighted the mitochondrial apoptotic signals role in the pathogenesis of acantholysis in PV, as well as of other autoimmune diseases.
- The mitochondrial voltage-dependent anion channel (VDAC) regulates apoptosis, but its role in PV remains unclear.

What does this study add?

- *VDAC1*, *VDAC2* and *VDAC3* are upregulated in ST18-overexpressing keratinocytes, with increased epidermal VDAC1 expression in PV skin.
- VDAC inhibition using the VDAC oligomerization inhibitor, VBIT-12, reduces apoptosis, increases Bcl-2 and downregulates p53 activity.
- VBIT-12 prevents PV IgG/AK23-induced acantholysis, highlighting VDAC as a novel factor in the pathogenesis of PV and thus as an innovative and attractive therapeutic target for the treatment of this disease.

Pemphigus vulgaris (PV) is a chronic autoimmune blistering disease that is most frequently diagnosed in individuals during their fifth to seventh decades of life.^1^ The use of corticosteroids and systemic immunosuppressants has led to a dramatic reduction in mortality rates to below 10%, with the majority of PV-related deaths being attributed to ­therapy-associated adverse effects.^1–3^ This underscores the need for innovative therapeutic strategies.

Circulating autoantibodies primarily directed against the desmosomal proteins desmoglein (Dsg)3 and Dsg1 have traditionally been considered the key factor in PV development.^1^ However, recent studies exploring PV pathogenesis have highlighted additional mechanisms, such as disrupted cell–cell signalling, apoptosis, the influence of ­proinflammatory cytokines and the activation of muscarinic receptors in keratinocytes.^4–9^ Moreover, it is well established that the tendency to develop PV is largely influenced by genetic factors.^10^ Over the past decade, ST18 has emerged as a key player in the pathogenesis of PV. *ST18* encodes a transcription factor that is overexpressed in the skin of patients with PV.^11,12^ A PV-associated risk variant within the *ST18* promoter region (rs17315309) was found to drive *ST18* transcription, stimulate PV serum-induced acantholysis and promote the release of key inflammatory molecules, particularly tumour necrosis factor alpha (TNF-α).^12–14^ Moreover, this effect of ST18 on cell–cell adhesion was found to be partially mediated by extracellular signal-regulated kinase signalling.^15^ Of note, the rs17315309 risk variant enhances ST18 expression in a p53-dependent manner,^13^ while depletion of Dsg3 in keratinocytes leads to increased p53 expression and activity.^16,17^ A recent study^14^ delineated a self-amplifying pathomechanism wherein ST18 expression accelerates autoantibody-driven Dsg3 membrane downregulation, which then activates p53, further regulating ST18.^13,14^

Previous data highlighted the role of mitochondrial apoptotic signals in the pathogenesis of acantholysis in PV, as well as of other autoimmune diseases, such as systemic lupus erythematosus (SLE).^18–25^ The voltage-dependent anion channel (VDAC) promotes the release of proapoptotic proteins from mitochondria.^26^ Notably, elevated expression of VDAC has also been observed in SLE,^27^ and inhibiting VDAC oligomerization has been shown to reduce disease severity in a mouse model of SLE.^28^ The fact that PV serum induces mitochondrial damage^20^ suggests the possibility of using mitochondria-protective drugs as a therapeutic approach in PV. Here we show that VDAC1 expression is increased in PV skin. Given that ST18 contributes to PV pathogenesis in part by inducing proapoptotic pathways, we aimed at investigating the interplay between ST18 and VDAC in apoptosis regulation, and the therapeutic potential of VDAC inhibitors in PV.

## Materials and methods

### Cell cultures

For the RNA sequencing (RNAseq) experiments, HaCaT cells were maintained in modified Eagle’s medium supplemented with 10% fetal calf serum, 1% L-glutamine, 1% streptomycin and 1% amphotericin (Biological Industries, Beit-Haemek, Israel) to assess ST18-dependent changes in *VDAC1*, *VDAC2* and *VDAC3*, and *BCL2* expression. For the caspase 3/7 and TUNEL apoptosis assays, the ­immunoblotting and a luciferase reporter assay to assess Bcl-2 and p53 pathways, and the dispase dissociation assay to test VDAC inhibition effect on acantholysis, normal human epidermal keratinocytes (NHEKs) were obtained from foreskin as previously described.^13^ The cells were grown in KC Growth Medium (Lonza, Walkersville, MD, USA). For expression studies, NHEKs grown to ∼70% confluence in 12-well plates were transfected with various constructs using Lipofectamine 2000 (Life Technologies, Carlsbad, CA, USA), as described elsewhere.^13^ Twenty-four hours post-transfection, NHEKs were exposed to the monoclonal antibody AK23 (3.75 μg mL^–1^; prior to exposure to AK23, Ca^2+^ concentration was raised to 1.2 mmol L^–1^) (D219-3; Biozol, Eching, Germany), a monoclonal mouse IgG1 antibody (3.75 μg mL^–1^) (MAB002; R&D Systems, Minneapolis, MN, USA), VBIT-12 [20 μmol L^–1^ (31445; Cayman Chemical, Ann Arbor MI, USA)] or dimethyl sulfoxide (DMSO) as a control. For caspase 3/7 assay and TUNEL staining, NHEKs were added at the same time as exposure to recombinant human TNF-α [20 ng mL^–1^ (300-01A; PeproTech, Cranbury, NJ, USA)] for 24 h.

### Monoclonal stable cell line generation

Human wildtype *ST18* open reading frame sequence cloned into a FLAG-tagged pCMV6-Entry vector (pCMV6-ST18) was purchased from Origene Technologies Company (Rockville, MD, USA). The empty pCMV6-Entry (EV) was used as a control.^13^ HaCaT cells were transiently transfected using Lipofectamine 2000 (Invitrogen, Grand Island, NY, USA) with pCMV6-ST18 or EV. The cells were cultured for another 2 weeks with media supplemented with G418. Monoclonal cell lines were generated by the limiting dilution method. Colonies were isolated and propagated. Monoclonal stable cell lines were validated through polymerase chain reaction (PCR) amplification of the expression vector.

### RNA sequencing

RNA was extracted using an RNA extraction kit (Roche, Mannheim, Germany). Libraries were prepared using a TrueSeq stranded total RNA LT sample prep kit (Illumina, San Diego, CA, USA). Sequencing was performed as a paired-end read on an Illumina True-Seq platform. Sequencing depth was ∼30 million reads/sample. Raw sequencing data were trimmed using fastp 0.20.0^29^ and aligned to the GRCm38 assembly using STAR 2.6.0c.^30^ DESeq2 1.30.1^31^ and R 3.6 were used for normalization of count data and for statistical analysis of differential gene expression. Cluster analysis was applied to the differentially expressed genes (DEGs) identified, and volcano maps were created for visualizing the data. Subsequently, Kyoto Encyclopedia of Genes and Genomes (KEGG) functional annotation, as well as functional enrichment analysis, were conducted to elucidate the functional and regulatory associations of the DEGs. Pathway analysis was used to identify genes involved in apoptosis, in part from the KEGG pathway (pathway ID: hsa04210). For quantitative reverse transcription PCR (qRT-PCR), RNA was extracted from cultured cells using an RNA extraction kit (Roche, Mannheim, Germany). cDNA was synthesized and PCR-amplified as described previously.^32^ Cycling conditions were as follows: 95 °C for 20 s, followed by 95 °C for 3 s and 60 °C for 30 s for 40 cycles. Primers used for the qRT-PCR experiments are listed in Table S1 (see Supporting Information). Each sample was analysed in triplicate. Results were normalized to *GAPDH* mRNA levels.

### Immunohistochemistry studies

After antigen retrieval with 0.01 mol L^–1^ citrate buffer (pH 6.0; Invitrogen, Carlsbad, CA, USA) in a microwave for 25 min, blocking with hydrogen peroxide for 10 min and protein blocking for 40 min, 5-mm-thick ­paraffin-embedded sections fixed on Plus glass slides (Menzel Glaser, Braunschweig, Germany) were processed with an automated immuno­stainer (Benchmark-XT; Ventana Medical System, Tucson, AZ, USA) with rabbit polyclonal anti-VDAC1/Porin antibody [diluted 1:200 (ab15895; Abcam, Cambridge, UK)]. Negative controls consisted of slides processed while omitting the primary antibody. Visualization of the bound primary antibodies was done with the Mouse and Rabbit Specific HRP/AEC Detection IHC Kit (ab93705; Abcam). Sections were then counterstained with Gill haematoxylin, dehydrated and mounted for microscopic examination. Specimens were examined with a Nikon 50I microscope connected to a DS-RI1 digital camera (Nikon, Tokyo, Japan).

### Immunofluorescence studies

NHEKs were grown on glass coverslips and fixed with paraformaldehyde 4%. Following permeabilization with Triton/phosphate-buffered saline 0.1%, sections were blocked in bovine serum albumin (BSA) 5% and incubated overnight at 4 °C with primary antibodies: rabbit polyclonal anti-VDAC1/Porin antibody [diluted 1:200; ab15895 (Abcam)] or mouse monoclonal anti-Bcl-2 Antibody (C-2) [diluted 1:50 (sc-7382; Santa Cruz Biotechnology, Santa Cruz, CA, USA)]. Secondary antibody staining was carried out for 1 h at 37 °C using goat antirabbit IgG (H + L) cross-adsorbed secondary antibody, Rhodamine Red™-X [diluted 1:200; #R-6394 (Invitrogen, Carlsbad, CA, USA)] or goat antimouse IgG (H + L) cross-­adsorbed secondary antibody, Rhodamine Red-X [diluted 1:200; #R-6393 (Invitrogen, Carlsbad, CA, USA)]. Negative control staining without the primary antibodies is shown in Figure S1 (see Supporting Information). Coverslips were mounted in polyvinyl alcohol. Imaging was done with Leica SP8 confocal microscope (Leica Microsystems, Wetzlar, Germany). Fluorescence intensity was quantified using ImageJ software (National Institutes of Health, Bethesda, MD, USA).

### Caspase 3/7 assay

The Caspase-Glo^®^ 3/7 Assay System kit (G8090; Promega, Madison, WI, USA) was conducted according to the manufacturer’s instructions. Briefly, cells were plated in 96-well white-walled, clear-bottom plates (Lonza, Basel, Switzerland). One hundred microlitres of the assay reagent was added to each well. The plate was then incubated for 30–60 min, and luminescence was measured using a Tecan plate reader (Tecan Group, Männedorf, Switzerland).

### TUNEL staining

The In Situ Cell Death Detection Kit, TMR red–TUNEL staining kit (12156792910; Roche) for apoptosis detection assay was used according to the instructions provided by the manufacturer. Fluorescence microscopy was done as described in the ‘Immunofluorescence studies’ section. Nuclei ­double-labelled with TUNEL and 4’,5-diamidino-2-phenyl­indole were considered indicative of apoptosis.

### p53 luciferase reporter assay

NHEKs cultured in a white flat-bottom 96-well microplate were transfected in the presence of Lipofectamine 2000 (Invitrogen, Grand Island, NY, USA) with a luciferase reporter construct containing an artificial p53-­responsive promoter (pRGC-luc) as previously described,^14^ or an empty luciferase pGL2 as a negative control, as well as a *Renilla* expression vector. Twenty-four hours post-­transfection, Ca^2+^ concentration was increased to 1.2 mmol L^–1^ and cells were treated with the monoclonal AK23 antibody [3.75 μg mL^–1^ (D219-3; Biozol)] or a monoclonal mouse IgG1 antibody [3.75 μg mL^–1^ (MAB002; R&D Systems)] as negative control, as well as VBIT-12 [20 μmol L^–1^ (31445; Cayman Chemical)] or DMSO as a control. Twenty-four hours later, a dual luciferase assay (Promega) was used to measure luciferase activity, which was normalized to *Renilla* luciferase activity, using a Tecan Infinite M200 device.

### Western blotting

As described elsewhere,^14^ cells were homogenized in CelLytic MT (Sigma-Aldrich, St. Louis, MO, USA) and a protease inhibitor mix, including 1 mmol L^–1^ phenylmethanesulfonyl fluoride, and 1 mg mL^–1^ aprotinin and leupeptin (Sigma-Aldrich). Following centrifugation, at 10 000*g* for 10 min at 4 °C, proteins were electrophoresed through a gradient Bio-Rad gel (4–20% Criterion™ TGX Stain-Free) and onto a polyvinylidene fluoride membrane (Trans-Blot; Bio-Rad, Hercules, CA, USA). After blocking for 1 h using 1× Tris-buffered saline with Tween-20 (50 mmol L^–1^ Tris, 150 mmol L^–1^ NaCl, 0.01% Tween 20) with BSA 5%, blots were incubated overnight at 4 °C with mouse monoclonal anti-Bcl-2 Antibody (C-2) [diluted 1:200 (sc-7382; Santa Cruz Biotechnology)]. The blots were washed five times for 5 min each with 1× Tris-buffered saline, 0.1% Tween 20 with BSA 1.5%. After incubation with horseradish peroxidase (HRP)-conjugated goat antimouse antibody [diluted 1:10 000 (115-035-003; Jackson ImmunoResearch Laboratories, West Grove, PA, USA)], and subsequent washings, proteins were detected using the EZ-ECL chemiluminescence detection kit (Biological Industries, Beit Haemek, Israel). To compare the amount of protein in different samples, we reprobed the blots using a mouse monoclonal anti-α-tubulin antibody [diluted 1:5000 (T9026; Sigma-Aldrich)] and secondary HRP-conjugated goat ­antimouse antibody [diluted 1:10 000 (115-035-003; Jackson ImmunoResearch Laboratories]. Protein levels were quantified by ImageJ software.

### Cytochrome C release assay

Following the manufacturer’s instructions, we isolated the mitochondrial fraction using the Cytochrome C Releasing Apoptosis Assay Kit (ab65311; Abcam).

### Dispase-based dissociation assay

The assay was conducted as detailed elsewhere.^13^ The fragment number was evaluated by two independent evaluators. NHEKs were grown to confluence in six-well plates, followed by exposure to AK23 (3.75 μg mL^–1^) or IgG1 antibody (3.75 μg mL^–1^), as well as VBIT-12 or DMSO as a control. NHEKs were additionally treated at the same time with ABT-199 [0.5 μmol L^–1^; HY15531S (MedChemExpress, Monmouth Junction, NJ, USA)] for 24 h. Prior to exposure to AK23, Ca^2+^ concentration was raised to 1.2 mmol L^–1^ .

### Statistical analysis

Comparisons of values between two groups were performed with an unpaired or paired Student’s *t*-test. When more than two groups were evaluated, one-way anova was performed. A value of *P* < 0.05 was considered statistically significant.

## Results

### Global RNAseq demonstrates increase in *VDAC1*, *VDAC2* and *VDAC3*, and decrease in *BCL2* expression upon *ST18* overexpression

**Figure 1:**
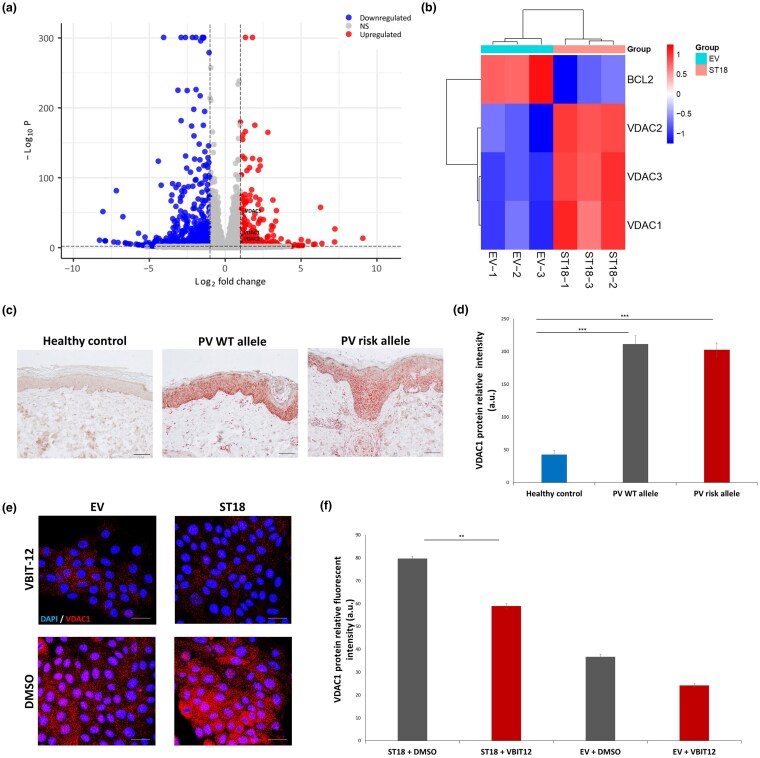
Voltage-dependent anion channel 1 (VDAC1) epidermal expression is increased in patients with pemphigus vulgaris (PV) and normal human epidermal keratinocytes (NHEKs) overexpressing ST18. (a) We used global RNA sequencing analysis to identify differentially expressed genes in HaCaT cells stably transfected with an *ST18*-expressing vector (ST18) vs. empty vector (EV). A volcano plot shows the comparative distribution of upregulated and downregulated genes in cells overexpressing *ST18* vs. control. Each point represents a gene, with the *x*-axis displaying the log2 fold change (|Log2 FC|) and the y-axis showing the –log10 of the *P*-value. Coloured dots indicate the categories of significance as follows: red dots and blue dots represent significantly upregulated and downregulated genes, respectively (*P* <0.01 and |Log2 FC| > 1). Genes not differentially expressed are coloured in grey (NS). (b) A heatmap plot of *VDAC1*, *VDAC2*, *VDAC3* and *BCL2* expression in three biological triplicates of each monoclonal HaCaT cell line (overexpressing *ST18*, right columns, or empty EV, left columns). Differential expression is shown as |Log2 FC|. (c) Immunohistochemistry of VDAC1 in skin biopsy samples obtained from healthy control participants (*n* = 5) or patients with PV carrying either the rs17315309 wildtype (WT; *n* = 5) or risk (*n* = 3) alleles (scale bar = 100 μm). (d) Expression of VDAC1 was quantified by ImageJ software (***P < 0.001 by two-tailed *t*-test). (e) NHEKs were transfected with an *ST18* expression vector (ST18) or with a control EV; 24 h post-transfection, cells were exposed to AK23 with VBIT-12 or dimethyl sulfoxide (DMSO) as a control for 24 h and were then fixed and immunostained for VDAC1 (red signal) and 4’,6-diamidino-2-phenylindole (DAPI; blue signal). (f) Expression of VDAC1 was quantified by ImageJ software. Results represent the mean (SE) of three independent experiments (**P < 0.01 by two-tailed *t*-test; scale bar = 20 μm). a.u., arbitrary units.

Given that ST18 has been shown to exacerbate PV IgG-induced epidermal intercellular disadhesion, in part by triggering proapoptotic pathways,^13,14^ we initially compared global gene expression in cells stably overexpressing ST18. We observed significant upregulation of *VDAC1*, *VDAC2* and *VDAC3*, which encode three subunits of VDAC (Figure 1a, b).^33–37^ VDAC is a known regulator of mitochondria-mediated apoptosis.^28,38^ Previous studies have implicated VDAC in PV pathogenesis.^21^ Additionally, *BCL2*, which encodes the antiapoptotic protein Bcl-2,^39^ was significantly downregulated in ST18-overexpressing cells compared with the EV control (Figure 1a, b). Bcl-2 is known to interact with VDAC, which inhibits its antiapoptotic activity^40^ and is also directly antagonized by p53.^41,42^ Gene expression changes were validated by qRT-PCR (Figure S2; see Supporting Information). Further pathway analysis, using the KEGG database, revealed that the DEGs in ST18-overexpressing HaCaT cells were predominantly associated with apoptotic signalling pathways and mitochondrial organization (Figure S3; see Supporting Information). This underscores the key role of ST18 in regulating apoptotic processes, possibly involving VDAC.

### VDAC1 expression is elevated in the skin of patients with PV and downregulated by VBIT-12 in NHEKs overexpressing ST18

To evaluate the clinical relevance of our findings, we performed immunohistochemistry staining for VDAC1 on nonlesional skin biopsies obtained from patients with PV and healthy control participants. Consistent with our RNAseq data, we observed significantly elevated VDAC1 expression in the epidermis of patients with PV, regardless of whether they carried the rs17315309 risk allele or the wildtype allele, as compared with healthy control participants (Figure 1c, d). VDAC2 and VDAC3 expression were not investigated due to lack of a specific antibody.

Next, we investigated the effect of ST18 on epidermal VDAC1 expression and its response to VBIT-12, a VDAC inhibitor, known to inhibit apoptosis and the infiltration of inflammatory cells.^43,44^ NHEKs were initially transfected with an ST18 expression vector or an EV as a control, followed by exposure to AK23, a pathogenic monoclonal antibody that targets the Dsg3 N-terminus and causes loss of epidermal cell–cell adhesion.^45^ The cells were treated with either VBIT-12 or DMSO as a control. Immunofluorescence staining revealed that NHEKs overexpressing ST18 showed a marked increase in VDAC1 expression vs. EV (Figure 1e, f). Furthermore, NHEKs overexpressing ST18 exhibited significantly reduced VDAC1 expression upon exposure to VBIT-12, as compared with NHEKs overexpressing ST18 and exposed to DMSO (Figure 1e, f). These results suggest that targeting VDAC with VBIT-12 may mitigate ST18-mediated apoptotic signalling.

### VBIT-12 attenuates AK23-induced apoptotic activity in NHEKs overexpressing ST18

**Figure 2:**
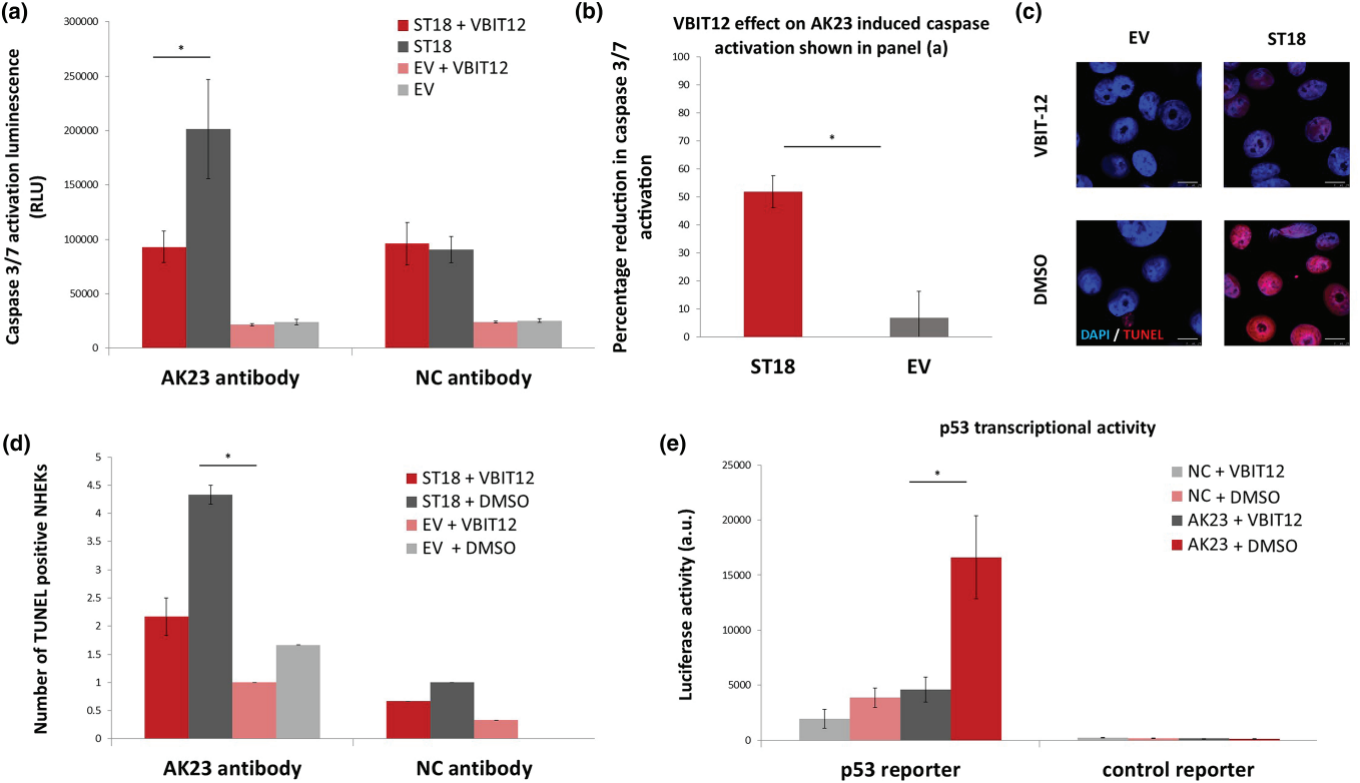
VBIT-12 attenuates AK23-induced apoptotic activity in normal human epidermal keratinocytes (NHEKs) overexpressing ST18. (a) NHEKs were transfected with an *ST18*-overexpressing vector (ST18) or with a control empty vector (EV); 24 h post-transfection cells were exposed to AK23 with VBIT-12 or dimethyl sulfoxide (DMSO) as a control, and were, at the same time, additionally exposed to recombinant human tumour necrosis factor alpha (20 ng mL^–1^) for 24 h. Caspase 3/7 activity was measured using the Caspase 3/7 Glo activity assay. Results represent the mean (SE) of three independent experiments (*P < 0.05 by two-tailed *t*-test). (b) The percentage reduction (%) in caspase 3/7 activation upon exposure to VBIT-12 compared with DMSO, in NHEKs exposed to AK23 and transfected with either an *ST18* expression vector (ST18) or with a control EV was calculated. Results represent the mean (SE) of three independent experiments (*P < 0.05 by two-tailed *t*-test). (c) Representative images of NHEKs that were fixed and immunostained for TUNEL activity (red signal) and 4’,6-diamidino-2-phenylindole (DAPI; blue signal) (scale bar = 10 μm). (d) TUNEL-positive NHEKs nuclei were quantified. Results represent the mean (SE) of three independent experiments (*P < 0.05 by two-tailed *t*-test). (e) NHEKs were transfected with a luciferase reporter construct under the regulation of a p53 binding site or with a control reporter. Twenty-four hours post-transfection, NHEKs were treated either with AK23 antibody or negative control (NC) antibody and exposed to VBIT-12 or DMSO. Results represent the mean (SE) of three independent experiments (*P < 0.05 by two-tailed *t*-test). a.u., arbitrary units; RLU, relative light unit.

**Figure 3:**
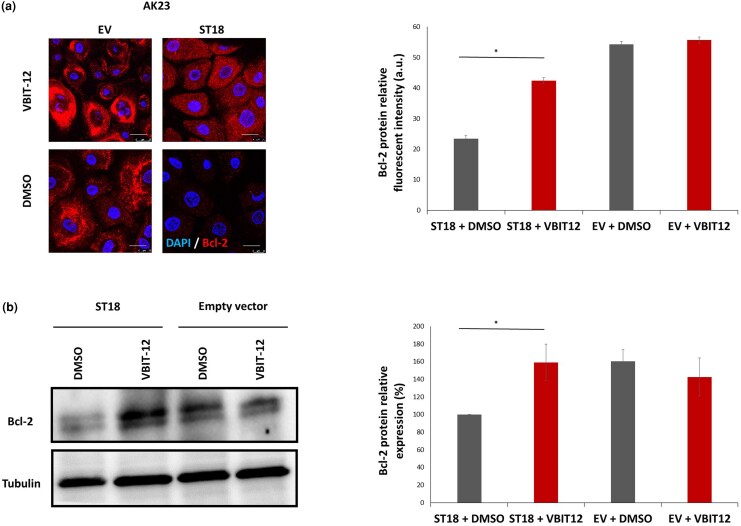
VBIT-12 upregulates the antiapoptotic protein Bcl-2 in normal human epidermal keratinocytes (NHEKs) overexpressing ST18 and exposed to AK23. (a) NHEKs were transfected with an *ST18* expression vector (ST18) or with a control empty vector (EV); 24 h post-transfection, cells were exposed to AK23 with VBIT-12 or dimethyl sulfoxide (DMSO) as a control for 24 h and were then fixed and immunostained for Bcl-2 (red signal) and 4’,6-diamidino-2-phenylindole (DAPI; blue signal) (left panel); expression of Bcl-2 was quantified by ImageJ software. Results represent the mean (SE) of three independent experiments (*P < 0.01 by two-tailed *t*-test; scale bar = 20 μm; right panel). (b) Bcl-2 protein expression was also assessed using immunoblotting with anti-Bcl-2 antibody. α-Tubulin served as a loading control (left panel). Protein levels were quantified, and data were normalized to levels observed in *ST18*-transfected cells treated with AK23 and DMSO. Results represent the mean (SE) of four independent experiments (*P < 0.05 by two-tailed *t*-test) (right panel). a.u., arbitrary units.

Given that the VDAC oligomerization inhibitor VBIT-12 has been proposed as a novel therapeutic option for ­autoimmune and chronic inflammatory diseases,^26,43^ we investigated its effect on AK23-induced apoptotic activity in ST18-overexpressing NHEKs. We ascertained apoptosis in cells transfected with either ST18 or EV and subsequently exposed to AK23, followed by treatment with VBIT-12 or DMSO as a control. Caspase 3/7 activity and TUNEL assays demonstrated significantly elevated apoptotic activity in NHEKs overexpressing ST18, compared with EV. Notably, treatment with VBIT-12 robustly attenuated this apoptotic response (Figure 2a–d). We next examined the effect of VBIT-12 on p53 activity using a luciferase reporter system under the regulation of a p53-binding motif, transfected into NHEKs. As shown in Figure 2(e), VBIT-12 significantly reduced AK23-induced p53 transcriptional activity compared with cells treated with DMSO, regardless of ST18 overexpression. Additionally, as Bcl-2 is known to interact with VDAC to inhibit its antiapoptotic function^40^ and is directly antagonized by p53,^41,42^ we further explored the effect of VBIT-12 on Bcl-2 expression levels in AK23-exposed ST18-overexpressing NHEKs using immunofluorescence staining, as well as western blotting. Cells overexpressing ST18 and exposed to AK23 exhibited significantly increased expression of the antiapoptotic protein Bcl-2 upon treatment with VBIT-12, as compared with cells treated with DMSO (Figure 3).

Furthermore, we investigated the release of cytochrome c, a proapoptotic protein released from mitochondria to the cytosol, which accelerates apoptosis.^46,47^ VDAC plays a role in mediating this release.^48^ NHEKs overexpressing ST18 and exposed to AK23 and VBIT-12 displayed a modest but statistically significant decrease in cytochrome c release from the mitochondria, as assessed with a Cytochrome C Releasing Apoptosis Assay Kit (Abcam), as compared with cells not exposed to VBIT-12 (Figure S4; see Supporting Information), which may be indicative of mitochondrial dysfunction. ST18 overexpression in the previous experiments was validated by qRT-PCR (Figure S5; see Supporting Information).

### VBIT-12 restores cell–cell adhesion stability in AK23-exposed normal human epidermal keratinocytes

**Figure 4:**
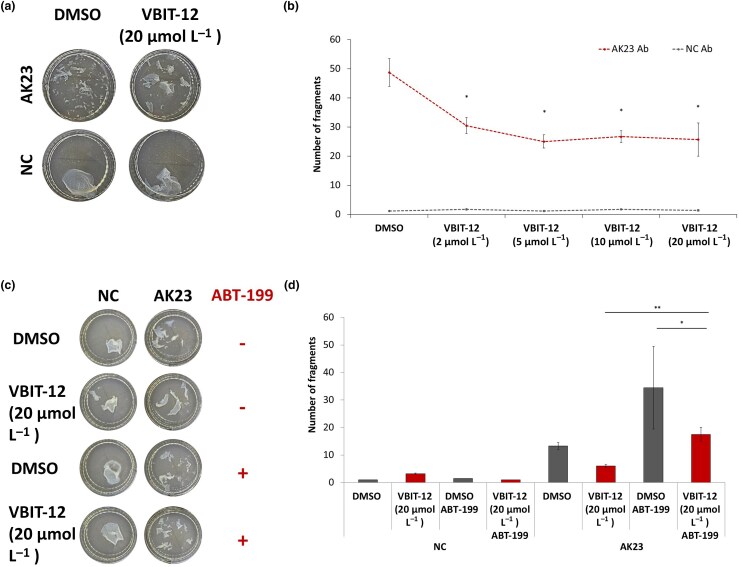
VBIT-12 restores cell–cell adhesion in AK23-exposed normal human epidermal keratinocytes (NHEKs). (a) NHEKs were grown to confluence, followed by exposure to AK23 or a negative control (NC), as well as different concentrations of VBIT-12 or dimethyl sulfoxide (DMSO) as a control. Epidermal sheets were then released from the tissue plates with dispase and subjected to mechanical stress. (b) Cell fragments were counted. Results represent the mean (SE) of three independent experiments (*P < 0.05 by two-tailed *t*-test). (c) NHEKs were grown to confluence, followed by exposure to AK23 or NC, as well as VBIT-12 or DMSO as a control, in the presence or absence of ABT-199 0.5 μmol L^–1^ . Epidermal sheets were then released from the tissue plates with dispase and subjected to mechanical stress. (d) Cell fragments were counted. Results represent the mean (SE) of three independent experiments (*P < 0.05, **P < 0.01 by two-tailed *t*-test).

Given the role of VDAC in apoptotic signalling and in PV pathogenesis, we investigated whether VBIT-12 could attenuate PV-related intraepidermal acantholysis. Using a ­dispase-based dissociation assay,^45^ we assessed intercellular adhesion in cultured NHEKs exposed to AK23 or a negative control antibody, followed by treatment with varying concentrations of VBIT-12 (0–20 μmol L^–1^) or DMSO as a control. Although not entirely linear, VBIT-12 significantly inhibited AK23-induced acantholysis in a dose-dependent manner (Figure 4a, b), supporting its therapeutic potential. As Bcl-2 interacts with VDAC to inhibit its antiapoptotic function,^40^ and VBIT-12 has been shown to increase Bcl-2 expression, we further explored the possibility that Bcl-2 may mediate, at least in part, the effect of VBIT-12 on AK23-induced acantholysis. We treated the cells with ABT-199, a potent and selective Bcl-2 inhibitor.^49,50^ Interestingly, ABT-199 attenuated the inhibitory effect of VBIT-12 to a significant extent (Figure 4c, d), suggesting that the inhibitory effect of VBIT-12 on AK23-induced acantholysis is mediated, at least in part, through Bcl-2, regardless of ST18 overexpression.

## Discussion

Advances in understanding PV pathogenesis, particularly the role of nonimmunological elements in blister formation, offer promising new directions for the treatment of this challenging disorder.^4,51^ Genetic and biological evidence have accumulated over the past decade pointing at the pivotal role of ST18 in PV pathogenesis.^11,13,15^ Here we showed that ST18 induces VDAC expression, a critical regulator of mitochondria-mediated apoptosis.^26^ As it is established that mitochondrial signalling is involved in apoptosis and PV,^19–22^ and as mitochondrial dysfunction has been shown to have a role in abnormal epidermal cell adhesion among patients with autoimmune bullous dermatoses,^18,23^ we attempted to target mitochondria-associated apoptosis as a new therapeutic strategy for PV.

VBIT-12, a potent VDAC1 inhibitor, has been shown to inhibit apoptosis and inflammation, and to preserve mitochondrial function.^43,44^ Accordingly, we demonstrate here that VBIT-12 downregulates VDAC1 expression (Figure 1e, f) and attenuates AK23-induced apoptotic activity in NHEKs overexpressing ST18 (Figure 2a–d). We also show that VBIT-12 significantly reduces AK23-induced p53 transcriptional activity (Figure 2e), which is required for the induction of apoptosis and ST18 upregulation.^14^ Interestingly, we have also shown that VBIT-12 dramatically increases the expression of the antiapoptotic protein Bcl-2 (Figure 3). This in line with the fact that Bcl-2 has been shown to interact with VDAC by inhibiting its antiapoptotic function,^40^ and is antagonized directly by p53.^41,42^ Moreover, VBIT-12 lead to a significant decrease (Figure S4) in protein cytochrome c^46,47^ release from the mitochondria, which is mediated by VDAC.^48^ Of note, the extent of cytochrome c release is strongly correlated with PV severity.^52^ These findings suggest that VBIT-12 effectively reduces apoptosis by modulating key apoptotic regulators such as p53, Bcl-2 and cytochrome c, suggesting it may have a therapeutic benefit through mitochondrial protection and apoptosis inhibition.

It is also important to emphasize that while the three VDAC isoforms (VDAC1, VDAC2 and VDAC3) identified in mammals share structural and functional features, they also exhibit distinct differences, including unique regulatory roles in cellular functions. Although VDAC1 is typically expressed at significantly higher levels than VDAC2 and VDAC3, we cannot rule out that the effects of VBIT-12 may involve VDAC2 and/or VDAC3.^53^ Recently, several potential nonimmunosuppressive treatments in PV, targeting distinct apoptotic pathways, were suggested, including soluble Fas ligand,^54^ caspase-3 signalling pathway inhibitors^55^ and MyD88 (which is downregulated by thalidomide).^56^ Accordingly, we demonstrated that VBIT-12 effectively reduces PV-related acantholysis, in part by modulating the Bcl-2-mediated pathway (Figure 4).

However, the present findings are primarily based on *in vitro* and *ex vivo* models, and their clinical relevance will require future validation in patients. Additionally, the ­off-target effects and safety profile of VBIT-12 were not assessed in this study and should be explored in future studies.

Overall, these findings delineate a novel pathomechanism underlying, in part, the detrimental effect of ST18 on cell–cell adhesion and suggesting the possibility of treating PV through VDAC inhibition.

## Supplementary Material

vzaf107_Supplementary_Data

## Data Availability

The data underlying this article will be shared on reasonable request to the corresponding author.
