# Supplementary material for: Targeting ST18-mediated pathomechanism in pemphigus vulgaris through voltage-dependent anion channel inhibition
Source: Skin Health Dis. 2026 Jan 19;6(2):150–9. doi: 10.1093/skinhd/vzaf107 (PMC13036731; doi:10.1093/skinhd/vzaf107)
Supplement: vzaf107_Supplementary_Data [file vzaf107_supplementary_data.docx]

**Supplementary data**

**Targeting ST18-mediated pathomechanism in pemphigus vulgaris through voltage-dependent anion channel inhibition**

**Running head:** Voltage-dependent anion channel Inhibition for ST18 mediated pemphigus vulgaris pathogenesis

Sari Assaf^1,2^, Ofer Sarig^1^, Rawaa Ishtewy^1,2^, Yazeed Zoabi^2^, Yarden Feller^1,2^, Kiril Malovitski^1,2^, Janan Mohamad^1,2^, Shir Bergson^1,2^, Carmel Bilu^1^, Varda Shoshan-Barmatz^3^, Noam Shomron^2^, Dan Vodo^1^, Liat Samuelov^1,2^, Eli Sprecher^1,2^

*^1^Division of Dermatology, Tel Aviv Sourasky Medical Center, Tel Aviv, Israel; ^2^Grey Faculty of Medicine and Health Sciences, Tel Aviv University, Tel Aviv, Israel; ^3^Division of Biotechnology, Ben-Gurion University of the Negev, Beer Sheva, Israel*

**Supplementary tables**

**Table S1: Sequence of oligonucleotides used for qRT-PCR**

| **Target gene** | **Forward oligonucleotide sequence** | **Reverse oligonucleotide sequence** |
| --- | --- | --- |
| *GAPDH* | GAGTCAACGGATTTGGTCGT | GACAAGCTTCCCGTTCTCAGCC |
| *ST18* | AAAACTCACGGGAAGACAGAG | GGTTTAGGGCTTGGTATAGAGG |
| *VDAC1* | GCAAAATCCCGAGTGACCCAGA | TCCAGGCAAGATTGACAGCGGT |
| *VDAC2* | CTTCTTACAAGAGGGAGTG | GTCCCATCATTGACATTAG |
| *VDAC3* | TCTGGACCAACCATCTA | AGGCTGGCATTATTTAC |
| *BCL2* | AACAGAGGGAGGGTTCCTGT | TTTTCCTCCCACCAGGTATG |

**Supplementary figures**

**Figure S1. Negative control for immunofluorescence staining**


As a negative control for IF studies, NHEKs were stained in the absence of a primary antibody with goat anti-rabbit IgG (H+L) cross-adsorbed secondary antibody, Rhodamine Red-X (diluted 1:200, Invitrogen, Carlsbad, CA, USA, #R-6394) **(a)** or goat anti-mouse IgG (H+L) cross-adsorbed secondary antibody, Rhodamine Red™-X (diluted 1:200, Invitrogen, Carlsbad, CA, USA, #R-6393) **(b)** (merged with DAPI, blue staining). (scale bar=10um).

**Figure S2: qRT-PCR validation of RNA-seq data**

Relative mRNA expression levels of *VDAC1-3, BCL2* (a) and *ST18* (b) in monoclonal HaCaT cell lines either overexpressing ST18 or an empty vector (EV) was ascertained by qRT-PCR. Results represent the mean (SE) of three independent experiments (* p<0.05, ***p<0.001 by 2-tailed t test).

**Figure S3: Pathway analysis of RNA-seq-derived differentially expressed genes (DEGs) in HaCaT cells stably transfected with *ST18* versus empty vector (EV)**


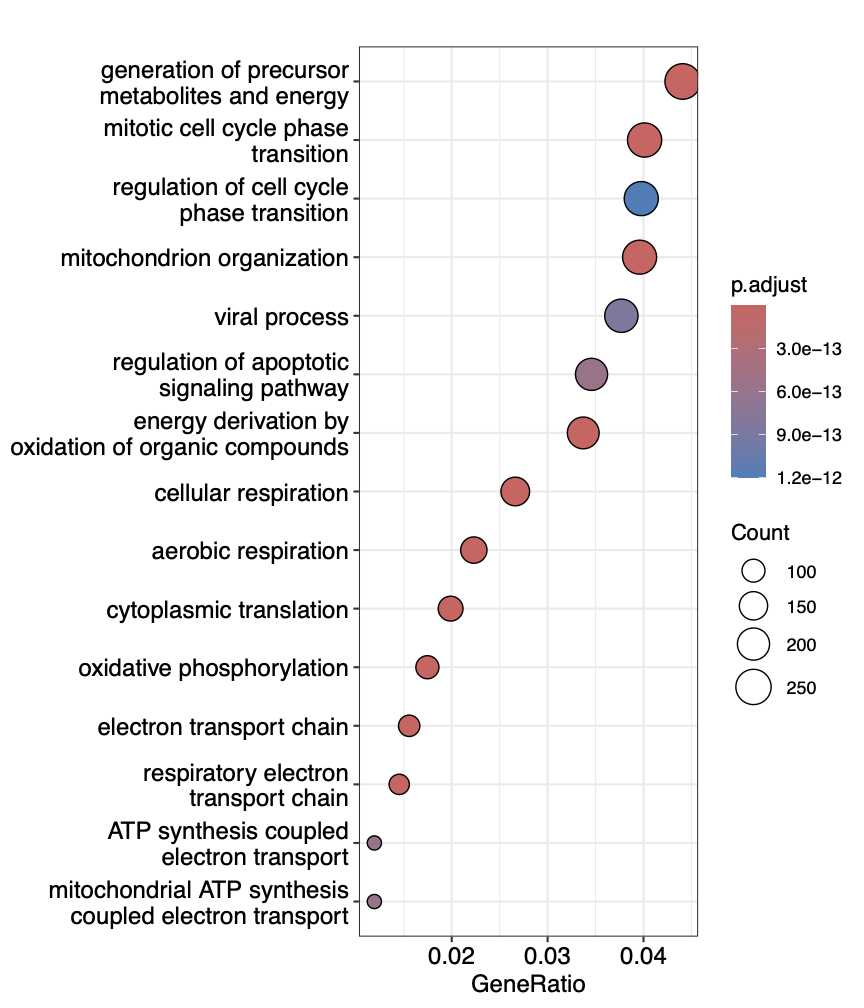


Kyoto Encyclopedia of Genes and Genomes (KEGG) bubble plot showing the top 15 enriched mRNAs in KEGG pathways. The bubble color represents the p-value, and the bubble size represents the number of genes in the relevant pathway (see plot legend).

**Figure S4: VBIT-12 inhibits cytochrome c release in NHEKs overexpressing ST18 and exposed to AK23**

NHEKs were transfected with an ST18 expression vector (ST18) or with a control empty vector (EV); 24 hours post transfection cells were exposed to AK23 with VBIT12 or DMSO as a control for 24 hours and then Cytochrome C release was measured using Cytochrome C releasing apoptosis assay kit (ab65311). **(a)** cytochrome c protein expression in cytosol was assessed using immunoblotting. α-tubulin served as a loading control; **(b)** Protein levels were quantified and data was normalized to levels observed in ST18-transfected cells treated with AK23 and DMSO. Results represent the mean + SE of three independent experiments (*p<0.05 by 2-tailed t test); **(c)** cytochrome c protein expression in mitochondria was also assessed using immunoblotting. α-tubulin served as a loading control; **(d)** Protein levels were quantified and data was normalized to levels observed in ST18-transfected cells treated with AK23 and DMSO. Results represent the mean (SE) of three independent experiments (*p<0.05 by 2-tailed t test).

**Figure S5: qRT-PCR validation of ST18 expression**

Relative mRNA expression levels of *ST18* in NHEKs either overexpressing ST18 or an empty vector (EV) was ascertained by qRT-PCR. Results represent the mean (SE) of three independent experiments (***p<0.001 by 2-tailed t test).
